# 1-Azido­ethoxy-2,3,4,6-tetra-*O*-acetyl-β-d-glucoside

**DOI:** 10.1107/S1600536809039737

**Published:** 2009-10-03

**Authors:** Xiao-Hui Yang, Yong-Hong Zhou, Hong-Jun Liu, Xiao-Xin Guo

**Affiliations:** a Institute of Chemical Industry of Forest Products, Chinese Academy of Forestry, Nanjing, 210042, People’s Republic of China

## Abstract

In the title compound, C_16_H_23_N_3_O_10_, the galactopyran­oside ring adopts a chair conformation. All the non-H substituents are situated in equatorial positions. There are short intramol­ecular C—H⋯O contacts and an intermolecular C—H⋯O inter­action in the structure.

## Related literature

For renewable compounds generated by living organisms that can be turned into useful macromolecular materials, see: Gandini (2008 ▶). For industrial applications of lignin, see: Gandini & Belgacem (2002 ▶). For attempts to obtain new polyurethanes between lignin and saccharide, see: Hatakeyama & Hatakeyama (2005 ▶).
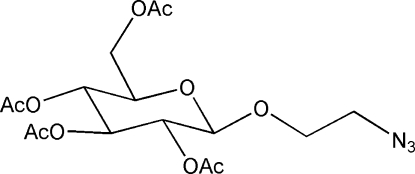


## Experimental

### 

#### Crystal data

C_16_H_23_N_3_O_10_
*M* *_r_* = 417.37
Orthorhombic, 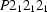

*a* = 6.9730 (14) Å
*b* = 14.747 (3) Å
*c* = 19.916 (4) Å
*V* = 2048.0 (7) Å^3^
*Z* = 4
Mo *K*α radiation
μ = 0.11 mm^−1^
*T* = 293 K
0.30 × 0.20 × 0.10 mm

#### Data collection

Enraf–Nonius CAD-4 diffractometer
Absorption correction: ψ scan (North *et al.*, 1968 ▶) *T*_min_ = 0.967, *T*_max_ = 0.989
3276 measured reflections
2152 independent reflections
1428 reflections with *I* > 2σ(*I*)
*R*_int_ = 0.036
3 standard reflections every 200 reflections intensity decay: 1%

#### Refinement

*R*[*F* ^2^ > 2σ(*F* ^2^)] = 0.055
*wR*(*F* ^2^) = 0.156
*S* = 1.06
2152 reflections
266 parameters
H-atom parameters constrained
Δρ_max_ = 0.24 e Å^−3^
Δρ_min_ = −0.20 e Å^−3^

### 

Data collection: *SMART* (Bruker, 2000 ▶); cell refinement: *SAINT* (Bruker, 2000 ▶); data reduction: *SAINT*; program(s) used to solve structure: *SHELXS97* (Sheldrick, 2008 ▶); program(s) used to refine structure: *SHELXL97* (Sheldrick, 2008 ▶); molecular graphics: *SHELXTL* (Sheldrick, 2008 ▶); software used to prepare material for publication: *SHELXTL*.

## Supplementary Material

Crystal structure: contains datablocks I, global. DOI: 10.1107/S1600536809039737/fb2162sup1.cif

Structure factors: contains datablocks I. DOI: 10.1107/S1600536809039737/fb2162Isup2.hkl

Additional supplementary materials:  crystallographic information; 3D view; checkCIF report

## Figures and Tables

**Table 1:** Hydrogen-bond geometry (Å, °)

*D*—H⋯*A*	*D*—H	H⋯*A*	*D*⋯*A*	*D*—H⋯*A*
C14—H14*A*⋯O4	0.98	2.21	2.666 (6)	107
C15—H15*A*⋯O2	0.98	2.32	2.723 (6)	104
C16—H16*A*⋯O7	0.98	2.27	2.702 (6)	106
C16—H16*A*⋯O9	0.98	2.44	2.824 (5)	103
C9—H9*B*⋯O1^i^	0.96	2.48	3.402 (6)	160

Symmetry code: (i) 
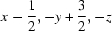
.

**Fig. 1.**
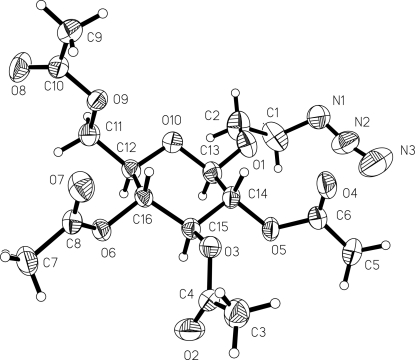
The title molecule with the atom-labelling scheme. The displacement ellipsoids drawn at the 30% probability level.

**Fig. 2.**
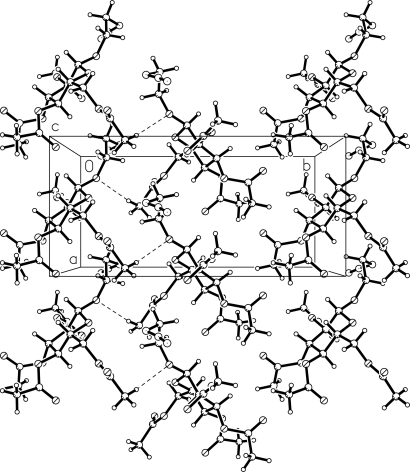
The packing of the title molecules, viewed along the c axis.

## supplementary crystallographic information

### Comment

The incessant biological activity in living organisms generates a multitude of compounds, including a variety of monomers and polymers such as saccharide, cellulose, hemicellulose, lignin and so on. More and more scientists are exclusively concerned with those renewable compounds that can be turned into useful macromolecular materials (Gandini, 2008). However, most of lignin as a by-product from the paper industry is being discharged into the environment. This causes serious environmental pollution. Also for this reason industrial applications of lignin have attracted a great deal of attention (Gandini & Belgacem, 2002).

In attempt to obtain new polyurethanes between lignin and saccharide (Hatakeyama & Hatakeyama, 2005) and to optimize their properties, the title structure, a new galactopyranoside, 2-azidoethoxy 2,3,4,6-tetra-*O*-acetyl-β-*D*-glucopyranoside, has been synthesized and its structure determined. The galactopyranoside ring adopts a chair conformation. There are present four intramolecular C-H···O interactionss (Tab. 1). Each of them  forms four five-membered rings. In the crystal structure, the molecules are linked into chains along the *a* axis by C—H···O interactions (Fig. 2 and Tab. 1).

### Experimental

β-D-glucose pentaacetate (5.0 g, 12.8 mmol) was dissolved in 25 ml of the anhydrous CH_2_Cl_2_. 2-azidoethanol (1.9 g, 22.3 mmol) was added to this solution by a syringe. The resulting solution was stirred under argon and cooled to 273 K. BF_3_.Et_2_O (2.1 ml, 16.7 mmol) was then added dropwise at 273 K. The mixture was stirred for 1 h at 273 K and then overnight at room temperature. The mixture was diluted with 50 ml CH_2_Cl_2_ and washed with cold water and with saturated aqueous NaHCO_3_ at room temperature, dried over anhydrous sodium sulfate, and concentrated *in vacuo* to obtain a fawn crude residue that was purified by column chromatography (hexane/EtOAc 2:1) and recrystallization from the solution of hexane/EtOAc (1:1) in order to obtain a pure solid of the title compound. Colourless single crystals suitable for X-ray crystallographic analysis were grown by slow evaporation from an ethyl acetate solution of the title compound.

### Refinement

All the H atoms were located in a difference electron density map. Nevertheless, all the hydrogens were placed into the idealized positions and constrained by riding hydrogen approximation. C_methyl_—H_methyl_=0.96; C_methylene_—H_methylene_=0.97; C_methine_—H_methine_=0.98 Å. U_iso_H_methyl_=1.5U_eq_C_methyl_, U_iso_H_methylene_=1.2U_eq_C_methylene_, U_iso_H_methine_=1.2U_eq_C_methine_. All the methyl groups were allowed to rotate freely about their respective C—C bonds during the refinement. Only 1/8 of the reciprocal space has been measured, therefore Friedel pairs for merging were not available.

### Crystal data

**Table:**

C_16_H_23_N_3_O_10_	*F*(000) = 880
*M**_r_* = 417.37	*D*_x_ = 1.354 Mg m^−^^3^
Orthorhombic, *P*2_1_2_1_2_1_	Mo *K*α radiation, λ = 0.71073 Å
Hall symbol: P 2ac 2ab	Cell parameters from 25 reflections
*a* = 6.9730 (14) Å	θ = 9–12°
*b* = 14.747 (3) Å	µ = 0.11 mm^−^^1^
*c* = 19.916 (4) Å	*T* = 293 K
*V* = 2048.0 (7) Å^3^	Block, colourless
*Z* = 4	0.30 × 0.20 × 0.10 mm

### Data collection

**Table:**

Enraf–Nonius CAD-4 diffractometer	1428 reflections with *I* > 2σ(*I*)
Radiation source: fine-focus sealed tube	*R*_int_ = 0.036
graphite	θ_max_ = 25.3°, θ_min_ = 1.7°
ω/2θ scans	*h* = −8→8
Absorption correction: ψ scan (North *et al.*, 1968)	*k* = 0→17
*T*_min_ = 0.967, *T*_max_ = 0.989	*l* = 0→23
3276 measured reflections	3 standard reflections every 200 reflections
2152 independent reflections	intensity decay: 1%

### Refinement

**Table:**

Refinement on *F*^2^	Primary atom site location: structure-invariant direct methods
Least-squares matrix: full	Secondary atom site location: difference Fourier map
*R*[*F*^2^ > 2σ(*F*^2^)] = 0.055	Hydrogen site location: difference Fourier map
*wR*(*F*^2^) = 0.156	H-atom parameters constrained
*S* = 1.06	*w* = 1/[σ^2^(*F*_o_^2^) + (0.0842*P*)^2^ + 0.0427*P*] where *P* = (*F*_o_^2^ + 2*F*_c_^2^)/3
2152 reflections	(Δ/σ)_max_ < 0.001
266 parameters	Δρ_max_ = 0.24 e Å^−^^3^
0 restraints	Δρ_min_ = −0.20 e Å^−^^3^
88 constraints	

### Special details

**Table:**

Geometry. All esds (except the esd in the dihedral angle between two l.s. planes) are estimated using the full covariance matrix. The cell esds are taken into account individually in the estimation of esds in distances, angles and torsion angles; correlations between esds in cell parameters are only used when they are defined by crystal symmetry. An approximate (isotropic) treatment of cell esds is used for estimating esds involving l.s. planes.
Refinement. Refinement of F^2^ against ALL reflections. The weighted R-factor wR and goodness of fit S are based on F^2^, conventional R-factors R are based on F, with F set to zero for negative F^2^. The threshold expression of F^2^ > 2sigma(F^2^) is used only for calculating R-factors(gt) etc. and is not relevant to the choice of reflections for refinement. R-factors based on F^2^ are statistically about twice as large as those based on F, and R- factors based on ALL data will be even larger.

### Fractional atomic coordinates and isotropic or equivalent isotropic displacement parameters (Å^2^)

**Table:**

	*x*	*y*	*z*	*U*_iso_*/*U*_eq_	
O1	0.7582 (5)	0.6069 (2)	0.05712 (18)	0.0722 (10)	
N1	1.0447 (9)	0.6905 (4)	0.1458 (3)	0.0961 (17)	
N2	1.0080 (9)	0.6436 (4)	0.1911 (4)	0.0987 (18)	
N3	0.9676 (16)	0.6099 (5)	0.2393 (4)	0.167 (3)	
O2	0.3076 (8)	0.2527 (2)	0.0360 (2)	0.1001 (14)	
C1	1.0868 (10)	0.6426 (5)	0.0830 (3)	0.103 (2)	
H1B	1.1128	0.5794	0.0929	0.123*	
H1C	1.2014	0.6684	0.0630	0.123*	
O3	0.2457 (5)	0.40003 (19)	0.05186 (15)	0.0560 (8)	
C2	0.9245 (9)	0.6483 (5)	0.0333 (3)	0.0917 (19)	
H2A	0.8978	0.7115	0.0237	0.110*	
H2B	0.9631	0.6193	−0.0083	0.110*	
O4	0.4451 (8)	0.5373 (3)	0.18472 (18)	0.0927 (13)	
C3	0.0437 (9)	0.2978 (4)	0.1058 (3)	0.0820 (17)	
H3A	0.0147	0.2344	0.1099	0.123*	
H3B	−0.0647	0.3290	0.0872	0.123*	
H3C	0.0724	0.3223	0.1493	0.123*	
O5	0.6040 (5)	0.4482 (2)	0.11247 (15)	0.0626 (9)	
C4	0.2113 (9)	0.3097 (3)	0.0612 (2)	0.0647 (13)	
O6	0.2735 (5)	0.40451 (19)	−0.09523 (15)	0.0554 (8)	
C5	0.6697 (10)	0.4265 (4)	0.2258 (3)	0.0854 (18)	
H5A	0.6339	0.4465	0.2700	0.128*	
H5B	0.8048	0.4357	0.2195	0.128*	
H5C	0.6403	0.3633	0.2210	0.128*	
O7	−0.0125 (5)	0.4706 (3)	−0.1036 (2)	0.0817 (11)	
C6	0.5624 (9)	0.4791 (3)	0.1751 (3)	0.0636 (13)	
O8	0.2457 (7)	0.6740 (3)	−0.23285 (18)	0.0903 (13)	
C7	0.0537 (10)	0.3357 (4)	−0.1658 (3)	0.0838 (18)	
H7A	−0.0777	0.3390	−0.1799	0.126*	
H7B	0.0760	0.2789	−0.1436	0.126*	
H7C	0.1360	0.3404	−0.2043	0.126*	
O9	0.2845 (5)	0.63294 (19)	−0.12588 (15)	0.0557 (8)	
C8	0.0953 (7)	0.4111 (3)	−0.1188 (2)	0.0541 (11)	
O10	0.5898 (5)	0.5863 (2)	−0.03885 (16)	0.0603 (8)	
C9	0.0336 (9)	0.7316 (3)	−0.1504 (3)	0.0789 (17)	
H9A	−0.0210	0.7666	−0.1863	0.118*	
H9B	0.0762	0.7716	−0.1154	0.118*	
H9C	−0.0614	0.6909	−0.1329	0.118*	
C10	0.1978 (8)	0.6792 (3)	−0.1759 (3)	0.0626 (13)	
C11	0.4448 (8)	0.5767 (3)	−0.1452 (2)	0.0636 (13)	
H11A	0.5502	0.6141	−0.1608	0.076*	
H11B	0.4076	0.5362	−0.1813	0.076*	
C12	0.5050 (7)	0.5231 (3)	−0.0851 (2)	0.0525 (11)	
H12A	0.6027	0.4791	−0.0987	0.063*	
C13	0.6755 (7)	0.5413 (3)	0.0177 (2)	0.0570 (12)	
H13A	0.7714	0.4970	0.0028	0.068*	
C14	0.5181 (7)	0.4956 (3)	0.0570 (2)	0.0520 (11)	
H14A	0.4290	0.5415	0.0740	0.062*	
C15	0.4101 (7)	0.4284 (3)	0.0141 (2)	0.0509 (11)	
H15A	0.4918	0.3761	0.0039	0.061*	
C16	0.3439 (6)	0.4733 (3)	−0.0504 (2)	0.0492 (11)	
H16A	0.2403	0.5161	−0.0403	0.059*	

### Atomic displacement parameters (Å^2^)

**Table:**

	*U*^11^	*U*^22^	*U*^33^	*U*^12^	*U*^13^	*U*^23^
O1	0.061 (2)	0.093 (2)	0.063 (2)	−0.014 (2)	0.0038 (19)	−0.021 (2)
N1	0.115 (4)	0.086 (3)	0.087 (4)	−0.004 (4)	−0.025 (4)	−0.003 (3)
N2	0.098 (4)	0.085 (4)	0.114 (5)	−0.005 (3)	−0.024 (4)	0.023 (3)
N3	0.200 (9)	0.142 (6)	0.158 (7)	0.013 (7)	−0.005 (8)	0.058 (6)
O2	0.148 (4)	0.0562 (19)	0.097 (3)	0.010 (3)	0.026 (3)	0.005 (2)
C1	0.064 (4)	0.126 (5)	0.117 (6)	−0.009 (4)	0.006 (4)	−0.040 (5)
O3	0.0622 (18)	0.0532 (16)	0.0526 (18)	0.0000 (16)	0.0079 (16)	−0.0005 (15)
C2	0.071 (4)	0.127 (5)	0.077 (4)	−0.024 (4)	0.013 (4)	0.001 (4)
O4	0.136 (4)	0.092 (3)	0.050 (2)	0.034 (3)	0.001 (2)	−0.016 (2)
C3	0.088 (4)	0.092 (4)	0.066 (4)	−0.022 (3)	−0.001 (3)	0.003 (3)
O5	0.073 (2)	0.075 (2)	0.0399 (17)	0.0134 (19)	−0.0103 (18)	−0.0057 (15)
C4	0.093 (4)	0.054 (3)	0.047 (3)	−0.005 (3)	−0.004 (3)	0.006 (2)
O6	0.065 (2)	0.0550 (16)	0.0466 (17)	0.0097 (16)	−0.0055 (17)	−0.0104 (15)
C5	0.109 (5)	0.097 (4)	0.050 (3)	0.000 (4)	−0.011 (3)	0.006 (3)
O7	0.062 (2)	0.098 (3)	0.085 (3)	0.011 (2)	−0.016 (2)	−0.014 (2)
C6	0.077 (3)	0.064 (3)	0.049 (3)	−0.002 (3)	−0.011 (3)	−0.008 (3)
O8	0.107 (3)	0.118 (3)	0.046 (2)	0.020 (3)	0.003 (2)	0.012 (2)
C7	0.106 (5)	0.090 (4)	0.055 (3)	−0.018 (4)	−0.007 (3)	−0.013 (3)
O9	0.062 (2)	0.0580 (16)	0.0475 (17)	0.0098 (16)	0.0027 (16)	0.0056 (15)
C8	0.053 (3)	0.066 (3)	0.044 (3)	0.002 (3)	0.000 (2)	−0.002 (2)
O10	0.0659 (19)	0.0599 (17)	0.0551 (19)	0.0019 (17)	−0.0010 (18)	0.0001 (16)
C9	0.094 (4)	0.072 (3)	0.071 (4)	0.018 (3)	−0.011 (3)	0.004 (3)
C10	0.074 (3)	0.059 (3)	0.054 (3)	−0.004 (3)	−0.012 (3)	0.003 (3)
C11	0.072 (3)	0.069 (3)	0.051 (3)	0.002 (3)	0.014 (3)	0.001 (2)
C12	0.057 (3)	0.053 (2)	0.048 (3)	0.006 (2)	0.002 (2)	−0.007 (2)
C13	0.053 (3)	0.065 (3)	0.054 (3)	0.003 (2)	−0.006 (2)	−0.009 (2)
C14	0.055 (3)	0.063 (3)	0.039 (2)	0.008 (2)	0.000 (2)	−0.002 (2)
C15	0.052 (3)	0.055 (2)	0.046 (2)	0.011 (2)	−0.005 (2)	−0.006 (2)
C16	0.053 (2)	0.051 (2)	0.043 (3)	0.012 (2)	−0.004 (2)	−0.010 (2)

### Geometric parameters (Å, °)

**Table:**

O1—C13	1.373 (5)	O7—C8	1.194 (5)
O1—C2	1.394 (7)	O8—C10	1.184 (6)
N1—N2	1.165 (7)	C7—C8	1.482 (6)
N1—C1	1.467 (7)	C7—H7A	0.9600
N2—N3	1.117 (9)	C7—H7B	0.9600
O2—C4	1.188 (6)	C7—H7C	0.9600
C1—C2	1.505 (9)	O9—C10	1.351 (6)
C1—H1B	0.9700	O9—C11	1.445 (6)
C1—H1C	0.9700	O10—C12	1.437 (5)
O3—C4	1.366 (5)	O10—C13	1.437 (5)
O3—C15	1.433 (5)	C9—C10	1.472 (7)
C2—H2A	0.9700	C9—H9A	0.9600
C2—H2B	0.9700	C9—H9B	0.9600
O4—C6	1.201 (6)	C9—H9C	0.9600
C3—C4	1.478 (8)	C11—C12	1.495 (6)
C3—H3A	0.9600	C11—H11A	0.9700
C3—H3B	0.9600	C11—H11B	0.9700
C3—H3C	0.9600	C12—C16	1.509 (6)
O5—C6	1.360 (6)	C12—H12A	0.9800
O5—C14	1.439 (5)	C13—C14	1.507 (7)
O6—C8	1.332 (6)	C13—H13A	0.9800
O6—C16	1.437 (5)	C14—C15	1.509 (6)
C5—C6	1.476 (8)	C14—H14A	0.9800
C5—H5A	0.9600	C15—C16	1.518 (6)
C5—H5B	0.9600	C15—H15A	0.9800
C5—H5C	0.9600	C16—H16A	0.9800
			
C13—O1—C2	117.6 (4)	C12—O10—C13	111.9 (3)
N2—N1—C1	114.7 (6)	C10—C9—H9A	109.5
N3—N2—N1	170.0 (9)	C10—C9—H9B	109.5
N1—C1—C2	112.5 (6)	H9A—C9—H9B	109.5
N1—C1—H1B	109.1	C10—C9—H9C	109.5
C2—C1—H1B	109.1	H9A—C9—H9C	109.5
N1—C1—H1C	109.1	H9B—C9—H9C	109.5
C2—C1—H1C	109.1	O8—C10—O9	123.2 (5)
H1B—C1—H1C	107.8	O8—C10—C9	125.8 (5)
C4—O3—C15	119.8 (4)	O9—C10—C9	111.0 (4)
O1—C2—C1	112.2 (5)	O9—C11—C12	107.9 (4)
O1—C2—H2A	109.2	O9—C11—H11A	110.1
C1—C2—H2A	109.2	C12—C11—H11A	110.1
O1—C2—H2B	109.2	O9—C11—H11B	110.1
C1—C2—H2B	109.2	C12—C11—H11B	110.1
H2A—C2—H2B	107.9	H11A—C11—H11B	108.4
C4—C3—H3A	109.5	O10—C12—C11	106.6 (3)
C4—C3—H3B	109.5	O10—C12—C16	109.2 (4)
H3A—C3—H3B	109.5	C11—C12—C16	114.5 (4)
C4—C3—H3C	109.5	O10—C12—H12A	108.8
H3A—C3—H3C	109.5	C11—C12—H12A	108.8
H3B—C3—H3C	109.5	C16—C12—H12A	108.8
C6—O5—C14	117.0 (4)	O1—C13—O10	107.3 (4)
O2—C4—O3	122.3 (5)	O1—C13—C14	108.9 (4)
O2—C4—C3	128.1 (5)	O10—C13—C14	108.1 (4)
O3—C4—C3	109.7 (5)	O1—C13—H13A	110.8
C8—O6—C16	119.1 (4)	O10—C13—H13A	110.8
C6—C5—H5A	109.5	C14—C13—H13A	110.8
C6—C5—H5B	109.5	O5—C14—C13	108.2 (4)
H5A—C5—H5B	109.5	O5—C14—C15	108.9 (3)
C6—C5—H5C	109.5	C13—C14—C15	111.3 (4)
H5A—C5—H5C	109.5	O5—C14—H14A	109.5
H5B—C5—H5C	109.5	C13—C14—H14A	109.5
O4—C6—O5	122.0 (5)	C15—C14—H14A	109.5
O4—C6—C5	127.7 (5)	O3—C15—C14	107.1 (3)
O5—C6—C5	110.1 (5)	O3—C15—C16	109.2 (4)
C8—C7—H7A	109.5	C14—C15—C16	110.1 (3)
C8—C7—H7B	109.5	O3—C15—H15A	110.1
H7A—C7—H7B	109.5	C14—C15—H15A	110.1
C8—C7—H7C	109.5	C16—C15—H15A	110.1
H7A—C7—H7C	109.5	O6—C16—C12	108.3 (3)
H7B—C7—H7C	109.5	O6—C16—C15	108.8 (3)
C10—O9—C11	116.1 (4)	C12—C16—C15	111.9 (4)
O7—C8—O6	123.5 (4)	O6—C16—H16A	109.3
O7—C8—C7	126.0 (5)	C12—C16—H16A	109.3
O6—C8—C7	110.5 (5)	C15—C16—H16A	109.3
			
C1—N1—N2—N3	−175 (5)	C6—O5—C14—C15	−125.1 (4)
N2—N1—C1—C2	104.9 (7)	O1—C13—C14—O5	−65.4 (5)
C13—O1—C2—C1	−125.6 (6)	O10—C13—C14—O5	178.3 (3)
N1—C1—C2—O1	−62.8 (8)	O1—C13—C14—C15	175.0 (4)
C15—O3—C4—O2	−4.1 (7)	O10—C13—C14—C15	58.7 (5)
C15—O3—C4—C3	175.6 (4)	C4—O3—C15—C14	−127.5 (4)
C14—O5—C6—O4	7.2 (7)	C4—O3—C15—C16	113.3 (4)
C14—O5—C6—C5	−176.9 (4)	O5—C14—C15—O3	70.1 (4)
C16—O6—C8—O7	−1.7 (7)	C13—C14—C15—O3	−170.6 (3)
C16—O6—C8—C7	177.9 (4)	O5—C14—C15—C16	−171.2 (3)
C11—O9—C10—O8	1.2 (7)	C13—C14—C15—C16	−52.0 (5)
C11—O9—C10—C9	178.3 (4)	C8—O6—C16—C12	−114.6 (4)
C10—O9—C11—C12	−173.2 (4)	C8—O6—C16—C15	123.6 (4)
C13—O10—C12—C11	−172.7 (4)	O10—C12—C16—O6	−174.5 (3)
C13—O10—C12—C16	63.1 (5)	C11—C12—C16—O6	66.0 (4)
O9—C11—C12—O10	−69.9 (5)	O10—C12—C16—C15	−54.6 (4)
O9—C11—C12—C16	51.0 (5)	C11—C12—C16—C15	−174.1 (4)
C2—O1—C13—O10	−72.1 (6)	O3—C15—C16—O6	−73.1 (4)
C2—O1—C13—C14	171.2 (4)	C14—C15—C16—O6	169.5 (3)
C12—O10—C13—O1	177.7 (3)	O3—C15—C16—C12	167.2 (3)
C12—O10—C13—C14	−65.0 (5)	C14—C15—C16—C12	49.9 (5)
C6—O5—C14—C13	113.7 (5)		

### Hydrogen-bond geometry (Å, °)

**Table:**

*D*—H···*A*	*D*—H	H···*A*	*D*···*A*	*D*—H···*A*
C14—H14A···O4	0.98	2.21	2.666 (6)	107
C15—H15A···O2	0.98	2.32	2.723 (6)	104
C16—H16A···O7	0.98	2.27	2.702 (6)	106
C16—H16A···O9	0.98	2.44	2.824 (5)	103
C9—H9B···O1^i^	0.96	2.48	3.402 (6)	160

Symmetry codes: (i) *x*−1/2, −*y*+3/2, −*z*.
